# Ethical and Scientific Considerations Regarding Animal Testing and Research

**DOI:** 10.1371/journal.pone.0024059

**Published:** 2011-09-07

**Authors:** Hope R. Ferdowsian, Nancy Beck

**Affiliations:** 1 Physicians Committee for Responsible Medicine, Washington, D.C., United States of America; 2 Department of Medicine, The George Washington University, Washington, D.C., United States of America; Public Library of Science, United Kingdom

In 1959, William Russell and Rex Burch published the seminal book, *The Principles of Humane Experimental Technique,* which emphasized reduction, refinement, and replacement of animal use, principles which have since been referred to as the “3 Rs”. These principles encouraged researchers to work to reduce the number of animals used in experiments to the minimum considered necessary, refine or limit the pain and distress to which animals are exposed, and replace the use of animals with non-animal alternatives when possible. Despite the attention brought to this issue by Russell and Burch and since, the number of animals used in research and testing has continued to increase, raising serious ethical and scientific issues. Further, while the “3 Rs” capture crucially important concepts, they do not adequately reflect the substantial developments in our new knowledge about the cognitive and emotional capabilities of animals, the individual interests of animals, or an updated understanding of potential harms associated with animal research. This Overview provides a brief summary of the ethical and scientific considerations regarding the use of animals in research and testing, and accompanies a Collection entitled *Animals, Research, and Alternatives: Measuring Progress 50 Years Later*, which aims to spur ethical and scientific advancement.

## Introduction

One of the most influential attempts to examine and affect the use of animals in research can be traced back to1959, with the publication of *The Principles of Humane Experimental Technique* [1]. William Russell and Rex Burch published this seminal book in response to marked growth in medical and veterinary research and the concomitant increase in the numbers of animals used. Russell and Burch's text emphasized reduction, refinement, and replacement of animal use, principles which have since been referred to as the “3 Rs”. These principles encouraged researchers to work to reduce the number of animals used in experiments to the minimum considered necessary, refine or limit the pain and distress to which animals are exposed, and replace the use of animals with non-animal alternatives when possible.

Despite the attention brought to this issue by Russell and Burch, the number of animals used in research and testing has continued to increase. Recent estimates suggest that at least 100 million animals are used each year worldwide [2]. However, this is likely an underestimate, and it is impossible to accurately quantify the number of animals used in or for experimentation. Full reporting of all animal use is not required or made public in most countries. Nevertheless, based on available information, it is clear that the number of animals used in research has not significantly declined over the past several decades.

The “3 Rs” serve as the cornerstone for current animal research guidelines, but questions remain about the adequacy of existing guidelines and whether researchers, review boards, and funders have fully and adequately implemented the “3 Rs”. Further, while the “3 Rs” capture crucially important concepts, they do not adequately reflect the substantial developments in our new knowledge about the cognitive and emotional capabilities of animals; an updated understanding of the harms inherent in animal research; and the changing cultural perspectives about the place of animals in society [3], [4]. In addition, serious questions have been raised about the effectiveness of animal testing and research in predicting anticipated outcomes [5]–[13].

In August 2010, the Georgetown University Kennedy Institute of Ethics, the Johns Hopkins University Center for Alternatives to Animal Testing, the Institute for In Vitro Sciences, The George Washington University, and the Physicians Committee for Responsible Medicine jointly held a two day multi-disciplinary, international conference in Washington, DC, to address the scientific, legal, and political opportunities and challenges to implementing alternatives to animal research. This two-day symposium aimed to advance the study of the ethical and scientific issues surrounding the use of animals in testing and research, with particular emphasis on the adequacy of current protections and the promise and challenges of developing alternatives to the use of animals in basic research, pharmaceutical research and development, and regulatory toxicology. Speakers who contributed to the conference reviewed and contributed new knowledge regarding the cognitive and affective capabilities of animals, revealed through ethology, cognitive psychology, neuroscience, and related disciplines. Speakers also explored the dimensions of harm associated with animal research, touching on the ethical implications regarding the use of animals in research. Finally, several contributors presented the latest scientific advances in developing alternatives to the use of animals in pharmaceutical research and development and regulatory toxicity testing.

This Collection combines some papers that were written following this conference with an aim to highlight relevant progress and research. This Overview provides a brief summary of the ethical and scientific considerations regarding the use of animals in research and testing, some of which are highlighted in the accompanying Collection.

## Analysis and Discussion

### Ethical Considerations and Advances in the Understanding of Animal Cognition

Apprehension around burgeoning medical research in the late 1800s and the first half of the 20^th^ century sparked concerns over the use of humans and animals in research [14], [15]. Suspicions around the use of humans were deepened with the revelation of several exploitive research projects, including a series of medical experiments on large numbers of prisoners by the Nazi German regime during World War II and the Tuskegee syphilis study. These abuses served as the impetus for the establishment of the Nuremberg Code, Declaration of Helsinki, and the National Commission for the Protection of Human Subjects of Biomedical and Behavioral Research (1974) and the resulting Belmont Report [16]–[18]. Today, these guidelines provide a platform for the protection of human research subjects, including the principles of respect, beneficence, and justice, as well as special protections for vulnerable populations.

Laws to protect animals in research have also been established. The British Parliament passed the first set of protections for animals in 1876, with the Cruelty to Animals Act [19]. Approximately ninety years later, the U.S. adopted regulations for animals used in research, with the passage of the Laboratory Animal Welfare Act of 1966 [20]. Subsequent national and international laws and guidelines have provided basic protections, but there are some significant inconsistencies among current regulations [21]. For example, the U.S. Animal Welfare Act excludes purpose-bred birds, rats, or mice, which comprise more than 90% of animals used in research [20]. In contrast, certain dogs and cats have received special attention and protections. Whereas the U.S. Animal Welfare Act excludes birds, rats and mice, the U.S. guidelines overseeing research conducted with federal funding includes protections for all vertebrates [22], [23]. The lack of consistency is further illustrated by the “U.S. Government Principles for the Utilization and Care of Vertebrate Animals Used in Testing, Research and Training” which stress compliance with the U.S. Animal Welfare Act and “other applicable Federal laws, guidelines, and policies” [24].

While strides have been made in the protection of both human and animal research subjects, the nature of these protections is markedly different. Human research protections emphasize specific principles aimed at protecting the interests of individuals and populations, sometimes to the detriment of the scientific question. This differs significantly from animal research guidelines, where the importance of the scientific question being researched commonly takes precedence over the interests of individual animals. Although scientists and ethicists have published numerous articles relevant to the ethics of animal research, current animal research guidelines do not articulate the rationale for the central differences between human and animal research guidelines. Currently, the majority of guidelines operate on the presumption that animal research should proceed based on broad, perceived benefits to humans. These guidelines are generally permissive of animal research independent of the costs to the individual animal as long as benefits seem achievable.

The concept of costs to individual animals can be further examined through the growing body of research on animal emotion and cognition. Studies published in the last few decades have dramatically increased our understanding of animal sentience, suggesting that animals' potential for experiencing harm is greater than has been appreciated and that current protections need to be reconsidered. It is now widely acknowledged by scientists and ethicists that animals can experience pain and distress [25]–[29]. Potential causes of harm include invasive procedures, disease, and deprivation of basic physiological needs. Other sources of harm for many animals include social deprivation and loss of the ability to fulfill natural behaviors, among other factors. Numerous studies have demonstrated that, even in response to gentle handling, animals can show marked changes in physiological and hormonal markers of stress [30].

Although pain and suffering are subjective experiences, studies from multiple disciplines provide objective evidence of animals' abilities to experience pain. Animals demonstrate coordinated responses to pain and many emotional states that are similar to those exhibited by humans [25], [26]. Animals share genetic, neuroanatomical, and physiological similarities with humans, and many animals express pain in ways similar to humans. Animals also share similarities with humans in genetic, developmental, and environmental risk factors for psychopathology [25], [26]. For example, fear operates in a less organized subcortical neural circuit than pain, and it has been described in a wide variety of species [31]. More complex markers of psychological distress have also been described in animals. Varying forms of depression have been repeatedly reported in animals, including nonhuman primates, dogs, pigs, cats, birds and rodents, among others [32]–[34]. Anxiety disorders, such as post-traumatic stress disorder, have been described in animals including chimpanzees and elephants [35], [36], [37].

In addition to the capacity to experience physical and psychological pain or distress, animals also display many language-like abilities, complex problem-solving skills, tool related cognition and pleasure-seeking, with empathy and self-awareness also suggested by some research. [38]–[44]. Play behavior, an indicator of pleasure, is widespread in mammals, and has also been described in birds [45], [46]. Behavior suggestive of play has been observed in other taxa, including reptiles, fishes and cephalopods [43]. Self-awareness, assessed through mirror self-recognition, has been reported for chimpanzees and other great apes, magpies, and some cetaceans. More recent studies have shown that crows are capable of creating and using tools that require access to episodic-like memory formation and retrieval [47]. These findings suggest that crows and related species display evidence of causal reasoning, flexible learning strategies, imagination and prospection, similar to findings in great apes. These findings also challenge our assumptions about species similarities and differences and their relevance in solving ethical dilemmas regarding the use of animals in research.

### Predictive Value of Animal Data and the Impact of Technical Innovations on Animal Use

In the last decade, concerns have mounted about how relevant animal experiments are to human health outcomes. Several papers have examined the concordance between animal and human data, demonstrating that findings in animals were not reliably replicated in human clinical research [5]–[13]. Recent systematic reviews of treatments for various clinical conditions demonstrated that animal studies have been poorly predictive of human outcomes in the fields of neurology and vascular disease, among others [7], [48]. These reviews have raised questions about whether human diseases inflicted upon animals sufficiently mimic the disease processes and treatment responses seen in humans.

The value of animal use for predicting human outcomes has also been questioned in the regulatory toxicology field, which relies on a codified set of highly standardized animal experiments for assessing various types of toxicity. Despite serious shortcomings for many of these assays, most of which are 50 to 60 years old, the field has been slow to adopt newer methods. The year 2007 marked a turning point in the toxicology field, with publication of a landmark report by the U.S. National Research Council (NRC), highlighting the need to embrace *in vitro* and computational methods in order to obtain data that more accurately predicts toxic effects in humans. The report, “Toxicity Testing in the 21^st^ Century: A Vision and a Strategy,” was commissioned by the U.S. Environmental Protection Agency, partially due to the recognition of weaknesses in existing approaches to toxicity testing [49]. The NRC vision calls for a shift away from animal use in chemical testing toward computational models and high-throughput and high-content *in vitro* methods. The report emphasized that these methods can provide more predictive data, more quickly and affordably than traditional *in vivo* methods. Subsequently published articles address the implementation of this vision for improving the current system of chemical testing and assessment [50], [51].

While a sea change is underway in regulatory toxicology, there has been much less dialogue surrounding the replacement of animals in research, despite the fact that far more animals are used in basic and applied research than in regulatory toxicology. The use of animals in research is inherently more difficult to approach systematically because research questions are much more diverse and less proscribed than in regulatory toxicology [52]. Because researchers often use very specialized assays and systems to address their hypotheses, replacement of animals in this area is a more individualized endeavour. Researchers and oversight boards have to evaluate the relevance of the research question and whether the tools of modern molecular and cell biology, genetics, biochemistry, and computational biology can be used in lieu of animals. While none of these tools on their own are capable of replicating a whole organism, they do provide a mechanistic understanding of molecular events. It is important for researchers and reviewers to assess differences in the clinical presentation and manifestation of diseases among species, as well as anatomical, physiological, and genetic differences that could impact the transferability of findings. Another relevant consideration is how well animal data can mirror relevant epigenetic effects and human genetic variability.

Examples of existing and promising non-animal methods have been reviewed recently by Langley and colleagues, who highlighted advances in fields including orthodontics, neurology, immunology, infectious diseases, pulmonology, endocrine and metabolism, cardiology, and obstetrics [52].

Many researchers have also begun to rely solely on human data and cell and tissue assays to address large areas of therapeutic research and development. In the area of vaccine testing and development, a surrogate *in-vitro* human immune system has been developed to help predict an individual's immune response to a particular drug or vaccine [53], [54]. This system includes a blood-donor base of hundreds of individuals from diverse populations and offers many benefits, including predictive high-throughput *in vitro* immunology to assess novel drug and vaccine candidates, measurement of immune responses in diverse human populations, faster cycle time for discovery, better selection of drug candidates for clinical evaluation, and reductions in the time and costs to bring drugs and vaccines to the market. In the case of vaccines, this system can be used at every stage, including *in vitro* disease models, antigen selection and adjuvant effects, safety testing, clinical trials, manufacturing, and potency assays. When compared with data from animal experiments, this system has produced more accurate pre-clinical data.

The examples above illustrate how innovative applications of technology can generate data more meaningful to humans, and reduce or replace animal use, but advances in medicine may also require novel approaches to setting research priorities. The Dr. Susan Love Research Foundation, which focuses on eradicating breast cancer, has challenged research scientists to move from animal research to breast cancer prevention research involving women. If researchers could better understand the factors that increase the risk for breast cancer, as well as methods for effective prevention, fewer women would require treatment for breast cancer. Whereas animal research is largely investigator-initiated, this model tries to address the questions that are central to the care of women at risk for or affected by breast cancer. This approach has facilitated the recruitment of women for studies including a national project funded by the National Institutes of Health and the National Institute of Environmental Health to examine how environment and genes affect breast cancer risk. This study, which began in 2002, could not have been accomplished with animal research [55].

Similarly, any approach that emphasizes evidence-based prevention would provide benefits to both animals and humans. Resource limitations might require a strategic approach that emphasizes diseases with the greatest public health threats, which increasingly fall within the scope of preventable diseases.

### Conclusion

It is clear that there have been many scientific and ethical advances since the first publication of Russell and Burch's book. However, some in the scientific community are beginning to question how well data from animals translates into germane knowledge and treatment of human conditions. Efforts to objectively evaluate the value of animal research for understanding and treating human disease are particularly relevant in the modern era, considering the availability of increasingly sophisticated technologies to address research questions [9]. Ethical objections to the use of animals have been publically voiced for more than a century, well before there was a firm scientific understanding of animal emotion and cognition [15]. Now, a better understanding of animals' capacity for pain and suffering is prompting many to take a closer look at the human use of animals [56].

Articles in the accompanying Collection only briefly touch on the many scientific and ethical issues surrounding the use of animals in testing and research. While it is important to acknowledge limitations to non-animal methods remain, recent developments demonstrate that these limitations should be viewed as rousing challenges rather than insurmountable obstacles. Although discussion of these issues can be difficult, progress is most likely to occur through an ethically consistent, evidence-based approach. This collection aims to spur further steps forward toward a more coherent ethical framework for scientific advancement.
